# Efficacy of vocal biomarkers in detecting cognitive impairment: interim findings from US and Indian populations

**DOI:** 10.1002/alz.086819

**Published:** 2025-01-09

**Authors:** Erik Larsen, Olivia Murton, Xinyu Song, Erica Weiss, Ayers Emmeline, Jessica Zwerling, Veena Sharath, Soumya Jacob, Alben Sigamani, V.G. Pradeep, Joe Verghese

**Affiliations:** ^1^ Sonde Health, Boston, MA USA; ^2^ Albert Einstein College of Medicine, Bronx, NY USA; ^3^ Montefiore Medical Center, Bronx, NY USA; ^4^ Department of neurology, Division of Cognitive and Motor Aging, Albert Einstein College of Medicine, New York, NY USA; ^5^ Research Consultancy, Bangalore, Karnataka India; ^6^ Baby Memorial Hospital, Kozhikode, Kerala India; ^7^ Department of Neurology, Division of Cognitive and Motor Aging, Albert Einstein College of Medicine, New York, NY USA

## Abstract

**Background:**

Vocal biomarkers are emerging as potentially meaningful health indicators in multiple domains, including cognition. Because voice‐enabled devices are widespread, automated vocal analysis could become a useful modality for early detection and monitoring of cognitive impairment. To assess the efficacy of vocal biomarkers in identifying cognitive impairment we evaluated prosodic speech features on vocal tasks in a research cohort from Kerala, India, and a referral cohort from the Montefiore‐Einstein Center for the Aging Brain in the Bronx, NY.

**Methods:**

157 participants (38% female, mean age 67 (4.9)) in India, and 57 participants (65% female, mean age 76 (6.3)) in NY completed voice protocols and cognitive screening (Addenbrooke's Cognitive Examination‐III in India, mean 81 (13); Blessed Information Memory Concentration Test in NY, mean 6.0 (3.7)). Prosodic speech features on vocal tasks with low and high mental effort were compared to performance on cognitive screening. Speech feature values were characterized using means and standard deviations, and subgroup distributions were compared using ratio of means, Cohen’s d for effect size, and significance testing using independent sample t‐tests.

**Results:**

Mean speech rate across conditions in syllables/minute was 100 (37) for the India and 107 (34) for the US cohort; pause duration in seconds was 0.68 (0.40) and 0.56 (0.33), respectively. Participants with worse performance on cognitive screening exhibited reduced speech rates and increased pause durations—23% variation in the Indian and 4% in the US cohort. Tasks with higher mental effort mirrored these effects, with about 25% variation in both cohorts. Mental effort had medium to large effect size (0.6‐0.9) and was statistically significant (p<0.01), while cognitive impairment achieved similar effect size and statistical significance for the India cohort only.

**Conclusion:**

These preliminary findings reinforce the potential of vocal biomarkers, especially speech rate and pause duration, as potential indicators of cognitive impairment in older adults. The results align with our goals to develop accessible vocal biomarker technology and support continued investigation into their utility in clinical and everyday settings, development of predictive analytics, and incorporating these into existing vocal biomarker platforms.

